# Associations between pesticide use and rheumatoid arthritis among older farmers in the Agricultural Health Study

**DOI:** 10.1038/s41598-024-76179-2

**Published:** 2024-12-02

**Authors:** Christine G. Parks, Darya Leyzarovich, Ghassan B. Hamra, Karen H. Costenbader, Dazhe Chen, Jonathan N. Hofmann, Laura E. Beane Freeman, Dale P. Sandler

**Affiliations:** 1 Epidemiology Branch, National Institute of Environmental Health Sciences, National Institutes of Health, Durham, NC USA; 2 DLH Corp., Bethesda, MD USA; 3 Division of Rheumatology, Inflammation and Immunity, Department of Medicine, Brigham and Women’s Hospital, Harvard Medical School, Boston, MA USA; 4 Occupational and Environmental Epidemiology Branch, Division of Cancer Epidemiology and Genetics, National Cancer Institute, Bethesda, MD USA

**Keywords:** Rheumatoid arthritis, Environmental sciences, Rheumatology, Risk factors

## Abstract

Pesticides and farming have been associated with increased rheumatoid arthritis (RA) risk, but the role of specific pesticides remains unknown. We examined RA risk among licensed pesticide applicators (97% white male farmers), from North Carolina and Iowa, in the Agricultural Health Study, in relation to lifetime use of 45 pesticides reported at enrollment (1993–1997, updated 1999–2003). In 22,642 applicators ages ≥ 67 years with ≥ 24 months Fee for Service Medicare data (1999–2016), we identified 161 (0.7%) incident cases with ≥ 2 RA claims (including ≥ 1 by a rheumatologist), ≥ 30 days apart, after ≥ 12 months without RA claims. Relative risks (RR) and 95% Confidence Intervals (CI) were calculated using log-binomial models adjusted for age, state, education, smoking, and correlated pesticides. Risk was elevated (RR > 1.5 or lower CI > 0.95) for use of nine pesticides: four insecticides [malathion (RR = 1.77;95%CI = 1.14–2.73), phorate (1.40;0.96–2.04), carbaryl (1.65;1.10–2.46), carbofuran (1.41;0.99–2.01)], four herbicides [alachlor (RR = 1.40;95%CI 0.99–1.98), metolachlor (1.57;1.11–2.23), S-Ethyl dipropylthiocarbamate (1.57;1.00-2.44), metribuzin (1.45; 1.01, 2.08)], and one fungicide [benomyl (1.56;0.99–2.44)]. Exposure-response was seen for greater intensity-weighted lifetime days use of malathion and carbofuran (p-trends = 0.03 and 0.05). Some specific pesticides, including several currently approved and commonly used in agricultural, public health, or residential settings may increase RA risk among older adults.

**Supplementary Information:**

The online version contains supplementary material available at 10.1038/s41598-024-76179-2.

## Background

Rheumatoid arthritis (RA) is a systemic autoimmune rheumatic disease (SARD) characterized by inflammation in the joints and effects in multiple organ systems, leading to long-term damage and disability^1^. Risk of RA is higher in older adults, and rates may be increasing worldwide, indicating a potential role of environmental factors^2,3^. Family history and genetics, cigarette smoking, and occupational exposure to respirable silica dust are the best-established environmental risk factors^4,5^. Farming occupation has been associated with RA in many but not all populations examined^6–10^, and some studies report associations with general pesticide use or classes of pesticides^7,11–13^. Pesticides include diverse active ingredients used as insecticides, herbicides, fungicides, and fumigants with varying mechanisms of action, and the role of specific pesticides in the development of RA remains unknown.

In the Agricultural Health Study (AHS), a prospective cohort of ~ 89,000 licensed pesticide applicators (mostly farmers) and spouses from North Carolina and Iowa enrolled in 1993–1997, previous research suggested some specific pesticides could be associated with self-reported RA^14,15^. In over 26,000 male private applicators completing at least one follow-up questionnaire (1999–2015; ~50% of those enrolled), 220 (0.8%) incident self-reported RA cases were identified and confirmed as probable cases based on use of disease-modifying anti-rheumatic medication and a limited number of medical records. Results showed associations of RA with the use of five specific pesticides reported at AHS enrollment, including 3 insecticides (toxaphene, fonofos, and carbary) and 2 herbicides (atrazine, and chlorimuron ethyl) with notable findings for two pesticides with widespread use, including a significant trend for increasing cumulative days mixing or applying atrazine, an agricultural herbicide, and a suggestive trend for carbaryl, an insecticide with residential and agricultural applications. Interpretation of these findings was limited by lack of specificity of self-reported RA^16^, and low participation in follow-up and disease validation^14^.

Here, we investigate the association of pesticides with RA risk in a sub-cohort of older AHS participants (ages ≥ 65 years, 1999–2016) linked to Medicare fee-for-service (FFS) administrative healthcare data^17^. These data provide an improved assessment of RA cases (including those who did not complete active follow-up) and identification of cases based on multiple medical claims for RA and specialist care by a rheumatologist. The study also updates data on pesticide use (prior to RA diagnosis) through 2003, and explores associations stratified by smoking, an established risk factor for RA^18^.

## Results

### Sample and case characteristics

Over a median of 8.5 (IQR 4.5–13.4) years of continuous FFS coverage we identified 22,084 participants without claims for RA, and 319 incident cases with ≥ 2 claims for RA ≥ 30 days apart, of which 161 were confirmed by specialist claims [median 4.4 (IQR1.8, 8.4) and 4.3 (1.9, 8.8) years to diagnosis, respectively Table 1]. Median age at the first RA claim was 73.7 (IQR 70.0, 77.9) and 72.9 (69.1, 76.9) years, respectively among overall and specialist-confirmed cases. Cases were older at AHS enrollment [median ages 61 (IQR 56, 67) overall, and 60 (IQR 55, 64) years specialist-confirmed), than non-cases [median 57 (IQR 51–64) years], were more often from North Carolina (54.9% overall, 54.0% specialist-confirmed, versus 40.8% non-cases), and fewer had a post-secondary education (21.9% overall and 26.1% specialist-confirmed, versus 31.7% non-cases). Cases were more likely to have smoked (overall, 60.2% and specialist-confirmed, 59.6%, versus 54.0% non-cases); median pack-years ranged from 0.5 (IQR 0.0, 15.0) in non-cases to 3.1 (0.0-22.5) in overall and 2.5 (0.0, 18.8) in specialist-confirmed cases. Overall cases had a similar lifetime days of general pesticide use (median 225; IQR = 64, 457) versus non-cases (225; 88–508); specialist-confirmed cases had fewer median days of use (179), but a similar IQR to overall and non-cases (64, 457). Main analyses were performed using specialist-confirmed cases, with ancillary analyses of overall cases, given differential access to specialist care among older participants in a rural environment^19^.

**Table 1:** Characteristics of AHS applicators with ≥ 24 months continuous Medicare-FFS: non-cases, possible and specialist-confirmed cases with incident rheumatoid arthritis

**Sample characteristics** ^**1**^		Cases	
	Non-cases	Overall^2^	Specialist-Confirmed^3^
	*N* = 22,084	*N* = 319	*N* = 161
Continuous FFS years (median, IQR)	8.5 (4.5, 13.4)	4.4 (1.8, 8.4)	4.3 (1.9, 8.8)
Diagnosis age (median, IQR)	---	73.7 (70.0, 77.9)	72.9 (69.1, 76.9)
**Enrollment**			
Age years (median, IQR)	57 (51, 64)	61 (56, 67)	60 (55, 64)
State (n, %)			
Iowa	13,063 (59.2%)	144 (45.1%)	74 (46.0%)
North Carolina	9021 (40.8%)	175 (54.9%)	87 (54.0%)
Education (n, %)			
≤High school	14,005 (63.4%)	230 (72.1%)	108 (67.1%)
> High school	6995 (31.7%)	70 (21.9%)	42 (26.1%)
Missing	1084 (4.9%)	19 (6.0%)	11 (6.8%)
Smoking (n, %)			
Ever	12,060 (54.0%)	194 (60.2%)	96 (59.6%)
Past	9360 (41.9%)	163 (50.6%)	77 (48.4%)
Current	2700 (12.1%)	31 (9.6%)	19 (11.8%)
Pack-years (median, IQR)	0.5 (0.0, 15.0)	3.1 (0.0, 22.5)	2.5 (0.0, 18.8)
Lifetime days mixed or applied pesticides (median, IQR)	225 (88, 508)	225 (64, 457)	179 (64, 457)

FFS, Fee for Service; IQR, cut-points for first and 3rd quartiles.

^1^Sample includes about 97% white males. The number of female and non-white cases, non-smokers, and missing smoking data are not shown, due to Ns < 11 or derivatives that may reveal number < 11 cases. Greater than high school education includes at least some college, associates, or 4-year college degree.

^2^Among participants with ≥ 24 months continuous FFS coverage (Parts A and B, without Part C), including an initial ≥ 12-month clean period with no claims for rheumatoid arthritis.

^3^Specialist-confirmed cases had ≥ 2 claims for RA ≥ 30 days apart plus ≥ 1 claims from a rheumatologist. Overall cases included all incident RA.

### Ever use of specific pesticides

Table 2 shows the frequency of 45 specific pesticides and results of log binomial regression models adjusted for age, state, education, smoking pack-years, and correlated pesticides (R2 > 0.35; Supplementary Table 1). Results showed 9 pesticides with elevated risk (i.e., RRs ≥ 1.5 and/or lower CIs ≥ 0.95): 4 insecticides [malathion (RR = 1.77;95%CI = 1.14–2.73), phorate (1.40;0.96–2.04), carbaryl (1.65;1.10–2.46), carbofuran (1.41;0.99–2.01)], 4 herbicides [alachlor (RR = 1.40;95%CI 0.99–1.98), metolachlor (1.57;1.11–2.23), S-Ethyl dipropylthiocarbamate (EPTC) (1.57;1.00-2.44), metribuzin (1.45; 1.01, 2.08)], and 1 fungicide [benomyl (1.56;0.99–2.44)].

**Table 2:** Ever use of specific pesticides and risk of specialist-confirmed RA among AHS applicators with ≥ 24 months continuous Medicare-FFS (1999–2016)

Ever-used^1^	Non-cases (*N* = 22,084)	Cases^2^ (*N* = 161)	Relative Risk (95% CI)^3^
	N (%)	N (%)	
**Insecticides**			
Organochlorines			
Aldrin	6502 (33)	46 (33)	1.07 (0.73–1.55)
Chlordane	7065 (36)	51 (36)	0.93 (0.66–1.32)
DDT	8906 (45)	72 (51)	1.05 (0.75–1.48)
Dieldrin	2295 (12)	14 (10)	0.85 (0.48–1.49)
Heptachlor	5178 (26)	33 (24)	0.98 (0.64–1.50)
Lindane	4674 (24)	33 (24)	1.09 (0.73–1.62)
Toxaphene	4169 (21)	40 (29)	1.31 (0.90–1.90)
Organophosphates			
Chlorpyrifos	8203 (41)	57 (40)	1.05 (0.75–1.48)
Diazinon	7059 (36)	53 (38)	0.99 (0.70–1.41)
Dichlorvos	2064 (11)	14 (10)	1.16 (0.66–2.04)
Fonofos	4328 (22)	31 (23)	1.37 (0.89–2.12)
Malathion	14,493 (73)	115 (82)	1.77 (1.14–2.73)
Parathion	3681 (19)	37 (26)	1.32 (0.90–1.94)
Phorate	7447 (38)	55 (39)	1.40 (0.96–2.04)
Terbufos	7122 (37)	50 (37)	1.25 (0.87–1.81)
Carbamates			
Aldicarb	2000 (10)	18 (13)	0.82 (0.47–1.45)
Carbaryl	12,247 (62)	106 (75)	1.65 (1.10–2.46)
Carbofuran	5917 (30)	49 (37)	1.41 (0.99–2.01)
Pyrethroids			
Permethrin - crops	2184 (11)	17 (13)	1.18 (0.71–1.96)
Permethrin - animals	1950 (10)	12 (9)	1.11 (0.61–2.03)
**Herbicides**			
Anilids/anilines			
Alachlor	10,517 (54)	81 (59)	1.40 (0.99–1.98)
Metolachlor	8647 (44)	70 (51)	1.57 (1.11–2.23)
Pendimethalin	8606 (44)	58 (41)	0.97 (0.69–1.36)
Trifluralin	10,103 (52)	70 (52)	1.29 (0.89–1.86)
Phenoxy			
2,4-D	15,885 (79)	109 (77)	1.08 (0.72–1.63)
2,4,5-T	6317 (32)	38 (27)	0.80 (0.54–1.17)
2,4,5-TP	2504 (13)	13 (9)	0.70 (0.40–1.25)
Thiocarbamate			
Butylate	6456 (34)	49 (36)	1.17 (0.79–1.74)
EPTC	3288 (17)	27 (20)	1.57 (1.00-2.44)
Triazine/Triazinone			
Atrazine	14,409 (74)	107 (78)	1.36 (0.88–2.12)
Cyanazine	7912 (41)	53 (38)	1.24 (0.84–1.82)
Metribuzin	8963 (46)	66 (47)	1.45 (1.01–2.08)
Other			
Chlorimuron Ethyl	6623 (34)	48 (34)	1.16 (0.82–1.66)
Dicamba	9581 (49)	60 (43)	1.12 (0.74–1.68)
Glyphosate	15,985 (80)	113 (80)	0.99 (0.65–1.50)
Imazethapyr	7492 (39)	44 (32)	1.07 (0.69–1.65)
Paraquat	4661 (24)	41 (29)	1.12 (0.77–1.65)
Petroleum Oil	8918 (46)	65 (46)	1.22 (0.86–1.72)
**Fungicides/Fumigants**			
Benomyl	2294 (12)	28 (20)	1.56 (0.99–2.44)
Captan	2054 (11)	16 (12)	1.15 (0.68–1.94)
Chlorothalonil	1583 (8)	20 (14)	1.32 (0.74–2.34)
Carbon tetrachloride/disulfide	1741 (9)	12 (9)	0.98 (0.54–1.78)
Maneb	2226 (11)	20 (14)	0.98 (0.59–1.62)
Metalaxyl	4547 (23)	44 (32)	1.25 (0.84–1.85)
Methyl bromide	3233 (16)	31 (22)	1.05 (0.67–1.65)

^1^Lifetime history ever used specific pesticides at enrollment (1993–1997) or first follow-up (1999–2003) prior to rheumatoid arthritis (RA) diagnosis.

^2^Among participants with ≥ 24 months continuous fee for service (FFS) coverage (Parts A and B without Part C), including an initial ≥ 12-month clean period with no claims for RA, specialist-confirmed cases had ≥ 2 claims for RA ≥ 30 days apart plus ≥ 1 claim from a rheumatologist.

^3^Relative Risk and 95% confidence intervals (CI) calculated using log binomial regression in covariate-adjusted models. Models adjust for categorical age at enrollment (40–49, 50–59, 60–69, 70+), state (NC, IA), education (≤ high school, > high school) and smoking pack-years, and correlated pesticides: aldicarb (chlorothalonil and benomyl), chlorothalonil (aldicarb and benomyl), atrazine (alachlor), butylate (cyanazine and metribuzin).

### Cumulative exposure-response

For 18 pesticides with ≥ 30% of cases exposed (i.e., 6 insecticides and 12 herbicides), we evaluated exposure-response trends for greater cumulative intensity-weighted lifetime days (i.e., IWLD: Tertiles, T1-3, and median split, M1-2, versus no use; in which T3 > T1 or M2 > M1; Table 3). Among the insecticides, we saw an exposure-response trend for malathion, in which the largest RR was seen for the top IWLD tertile (T1, 1.67;0.95–2.94, p_trend_=0.03)]. For carbaryl, the largest RR was seen for the lowest IWLD tertile (T1, 1.75; 0.97–3.16]. while for carbofuran, the largest RR was seen above the median IWLD (M2, 1.53; 0.99, 2.37; p_trend_=0.05). Among the herbicides, we saw no evidence of significant exposure-response trends. For alachlor, the largest RR was seen for the middle tertile of IWLD (T2 1.45; 0.91–2.30); and, while the trend p-value was 0.04 for metolachlor, RR were elevated for both the lowest and highest tertiles [(T1, 1.87;1.17-3.00) and (T3, 1.60;0.98–2.59)]. We saw an elevated RR for higher levels of butylate, but not a significant trend (M2, 1.74; 0.97,3.11; p_trend_=0.16). Atrazine showed no evidence of exposure-response (range of RRs Q1, 1.20 to Q4, 1.33; p_trend_=0.31), while the larger RR for metribuzin was below the median IWLD (M1, 1.82; 1.03–3.21).

**Table 3:** Intensity weighted lifetime days of pesticide use and risk of specialist-confirmed RA among AHS applicators with ≥ 24 months continuous FFS Medicare coverage (1999–2016).

**IWLD categories** ^**1**^	Non-cases	Cases^2^	Relative Risk
	(*N* = 22,084) N (%)	(*N* = 161) N (%)	**(95% CI)** ^**3**^
**Insecticides**			
Organochlorine			
DDT*			
None	6161 (63)	46 (60)	Reference
M1	1784 (18)	16 (21)	1.01 (0.57–1.80)
M2	1796 (18)	15 (19)	0.85 (0.47–1.54)
*p-trend*			0.63
Organophosphate			
Chlorpyrifos			
None	10,333 (56)	72 (56)	Reference
M1	3999 (22)	24 (19)	0.92 (0.58–1.45)
M2	3990 (22)	32 (25)	1.23 (0.81–1.86)
*p-trend*			0.42
Malathion*			
None	3193 (31)	22 (*)	Reference
T1	2418 (23)	< 11	0.55 (0.25–1.25)
T2	2370 (23)	18 (*)	1.19 (0.64–2.24)
T3	2378 (23)	28 (*)	1.67 (0.95–2.94)
*p-trend*			*0.03*
Terbufos			
None	10,886 (61)	75 (63)	Reference
M1	3437 (19)	18 (15)	0.97 (0.57–1.64)
M2	3429 (19)	27 (23)	1.39 (0.89–2.18)
*p-trend*			0.19
Carbamates			
Carbaryl*			
None	5205 (49)	31 (38)	Reference
T1	1803 (17)	18 (22)	1.75 (0.97–3.16)
T2	1810 (17)	15 (18)	1.35 (0.71–2.58)
T3	1802 (17)	18 (22)	1.36 (0.69–2.69)
*p-trend*			0.34
Carbofuran			
None	11,958 (68)	75 (61)	Reference
M1	2827 (16)	20 (16)	1.26 (0.77–2.08)
M2	2824 (16)	27 (22)	1.53 (0.99–2.37)
*p-trend*			0.05
**Herbicides**			
Anilids/anilines			
Alachlor			
None	7700 (43)	51 (41)	Reference
T1	3334 (19)	22 (18)	1.18 (0.71–1.96)
T2	3346 (19)	28 (23)	1.45 (0.91–2.30)
T3	3342 (19)	23 (19)	1.11 (0.68–1.81)
*p-trend*			0.38
Metolachlor			
None	9493 (54)	59 (46)	Reference
T1	2742 (15)	26 (20)	1.87 (1.17-3.00)
T2	2747 (16)	19 (15)	1.38 (0.82–2.33)
T3	2725 (15)	23 (18)	1.60 (0.98–2.59)
*p-trend*			0.04
Pendimethalin*			
None	6270 (61)	52 (*)	Reference
M1	1996 (19)	< 11	0.47 (0.21–1.04)
M2	1991 (19)	19 (*)	1.18 (0.70-2.00)
*p-trend*			0.92
Trifluralin			
None	8003 (45)	56 (46)	Reference
T1	3237 (18)	19 (16)	1.06 (0.62–1.80)
T2	3214 (18)	21 (17)	1.27 (0.75–2.14)
T3	3240 (18)	25 (21)	1.46 (0.89–2.38)
*p-trend*			0.12
Phenoxy			
2,4-D			
None	4034 (21)	32 (23)	Reference
Q1	3870 (20)	29 (21)	1.07 (0.65–1.78)
Q2	3878 (20)	23 (17)	0.92 (0.53–1.60)
Q3	3878 (20)	23 (17)	0.92 (0.53–1.61)
Q4	3866 (20)	30 (22)	1.19 (0.71-2.00)
*p-trend*			0.71
Thiocarbamate			
Butylate*			
None	7051 (73)	54 (*)	Reference
M1	1327 (14)	< 11	0.52 (0.20–1.33)
M2	1315 (14)	17 (*)	1.74 (0.97–3.11)
*p-trend*			0.16
Triazine/Triazinone			
Atrazine			
None	5027 (26)	31 (23)	Reference
Q1	3506 (18)	23 (17)	1.20 (0.69–2.11)
Q2	3513 (18)	26 (20)	1.34 (0.77–2.33)
Q3	3507 (18)	25 (19)	1.30 (0.74–2.28)
Q4	3506 (18)	27 (20)	1.33 (0.76–2.32)
*p-trend*			0.31
Cyanazine			
None	10,157 (57)	77 (61)	Reference
M1	3827 (21)	25 (20)	1.18 (0.73–1.92)
M2	3825 (21)	25 (20)	1.19 (0.74–1.94)
*p-trend*			0.43
Metribuzin*			
None	6061 (61)	48 (*)	Reference
M1	1926 (19)	20 (*)	1.82 (1.03–3.21)
M2	1946 (20)	< 11	0.89 (0.44–1.80)
*p-trend*			0.83
Other			
Dicamba			
None	8472 (48)	72 (56)	Reference
T1	3069 (17)	22 (17)	1.21 (0.71–2.06)
T2	3055 (17)	19 (15)	1.06 (0.61–1.86)
T3	3067 (17)	16 (12)	0.89 (0.49–1.60)
*p-trend*			0.75
Glyphosate			
None	4031 (21)	30 (21)	Reference
Q1	3857 (20)	24 (17)	0.84 (0.49–1.45)
Q2	3856 (20)	22 (16)	0.77 (0.44–1.34)
Q3	3857 (20)	28 (20)	0.99 (0.59–1.67)
Q4	3844 (20)	37 (26)	1.27 (0.77–2.08)
*p-trend*			0.25
Imazethapyr			
None	10,811 (60)	83 (66)	Reference
M1	3645 (20)	25 (20)	1.27 (0.75–2.13)
M2	3648 (20)	17 (14)	0.92 (0.51–1.64)
*p-trend*			0.92

^1^As reported in 1993-97 and updated 1999–2003. Scores are grouped based on percent exposed cases: median (30 < 50%), tertiles (50 < 70%), or quartiles (≥ 70%) as intensity-weighted lifetime days (IWLD) use of specific pesticides up through enrollment (1993–1997) or first follow-up (1999–2003) prior to RA diagnosis; *Enrollment IWLD based on the take home questionnaire.

^2^Among participants with ≥ 24 months continuous fee for service (FFS) coverage (Parts A and B without Part C), including an initial ≥ 12-month clean period with no RA claims; specialist-confirmed cases had ≥ 2 claims for RA ≥ 30 days apart, plus ≥ 1 claim from a rheumatologist. Asterix (*) indicates percent not shown if number or percent not shown due to < 11 in a cell or ability to infer the count of groups with < 11 exposed cases.

^3^Relative Risk (RR) and 95% Confidence Intervals (CI) calculated using log binomial regression. Models adjust for categorical age at enrollment (40–49, 50–59, 60–69, 70+), state (NC, IA) state, (NC, IA)-education (≤ high school, > high school), smoking pack years, and correlated pesticides (R2 > 0.350) [aldicarb (chlorothalonil and benomyl)-chlorothalonil (aldicarb and benomyl)-atrazine (alachlor)-butylate (cyanazine and metribuzin)].

### Smoking stratified analyses

For 39 pesticides with ≥ 11 exposed cases in both smokers and non-smokers (Table 4), we explored possible differences in smoking-stratified models. Among smokers, we observed associations (i.e., RRs ≥ 1.5 and/or lower CI ≥ 0.95) for 11 pesticides (phorate, carbaryl, carbofuran, alachlor, metolachlor, trifluralin, EPTC, atrazine, cyanazine, chlorimuron ethyl, and benomyl) and 3 among non-smokers (malathion, carbaryl, benomyl). Most interactions were not significant (*p* < 0.10), except for carbofuran [(RR = 1.74;95%CI 1.13–2.71) smokers, (0.89;0.49–1.62) non-smokers; _int_*p*=0.07)] and alachlor [(1.84;1.19–2.87) smokers, (0.96;0.55–1.67 non-smokers; _int_*p*=0.08)]. Among non-smokers, the RR for malathion was greater (RR 2.41;1.13, 5.18) than among smokers (1.36;0.82, 2.27), but the interaction was not significant (_int_*p*=0.28).

**Table 4:** Specific pesticide use in AHS applicators with ≥24 months continuous FFS Medicare coverage (1999-2016): risk of specialist-confirmed RA, stratified by smoking history (ever).

Ever used^1^:	% exposed cases^2^	Relative Risk (95% Confidence Intervals)^3^	
	Smokers / Non-smokers	Smokers	Non-smokers
**Insecticides**			
Organochlorines			
Aldrin^4^	35 / 28	1.30 (0.82–2.08)	0.75 (0.40–1.41)
Chlordane	34 / 37	0.82 (0.53–1.28)	1.07 (0.61–1.88)
DDT	54 / 48	1.02 (0.67–1.56)	1.06 (0.61–1.85)
Heptachlor	25 / 22	1.20 (0.70–2.06)	0.77 (0.39–1.53)
Lindane	22 / 22	1.08 (0.65–1.80)	0.94 (0.49–1.81)
Toxaphene	31 / 24	1.36 (0.86–2.14)	1.19 (0.64–2.24)
Organophosphates			
Chlorpyrifos	43 / 40	1.20 (0.79–1.83)	1.05 (0.61–1.82)
Diazinon	40 / 34	1.02 (0.66–1.58)	0.93 (0.52–1.67)
Fonofos	21 / 25	1.38 (0.78–2.44)	1.27 (0.65–2.48)
Malathion	78 / 85	1.36 (0.82–2.27)	2.41 (1.13–5.18)
Parathion	27 / 24	1.19 (0.74–1.92)	1.43 (0.75–2.73)
Phorate	42 / 39	1.80 (1.13–2.89)	1.12 (0.62–2.03)
Terbufos	36 / 38	1.30 (0.82–2.07)	1.13 (0.63–2.03)
Carbamates			
Aldicarb	16 / *	0.89 (0.46–1.72)	0.77 (0.27–2.19)
Carbaryl	77 / 72	1.53 (0.92–2.55)	1.77 (0.95–3.31)
Carbofuran^4^	40 / 28	1.75 (1.13–2.71)	0.89 (0.49–1.62)
**Herbicides**			
Analids/analines			
Alachlor^4^	64 / 42	1.84 (1.19–2.87)	0.96 (0.55–1.67)
Metolachlor	49 / 50	1.58 (1.02–2.45)	1.37 (0.79–2.38)
Pendimethalin	46 / 37	1.13 (0.74–1.72)	0.84 (0.48–1.47)
Trifluralin	53 / 47	1.50 (0.95–2.38)	0.90 (0.51–1.62)
Phenoxy			
2,4-D	39 / 30	1.26 (0.75–2.14)	0.83 (0.44–1.57)
2,4,5-T	28 / 24	0.84 (0.53–1.36)	0.66 (0.35–1.26)
Thiocarbamate			
Butylate^4^	39 / 30	1.42 (0.87–2.30)	0.84 (0.44–1.60)
EPTC	20 / *	1.70 (0.97–2.98)	1.31 (0.64–2.68)
Triazine/Triazinone			
Atrazine^4^	81 / 73	1.64 (0.93–2.92)	1.04 (0.52–2.06)
Cyanazine	40 / 38	1.54 (0.95–2.51)	0.97 (0.52–1.79)
Metribuzin	45 / 50	1.43 (0.90–2.26)	1.44 (0.81–2.57)
Other			
Chlorimuron Ethyl^4^	39 / 28	1.46 (0.95–2.25)	0.82 (0.45–1.49)
Dicamba	39 / 49	1.03 (0.61–1.73)	1.12 (0.59–2.11)
Glyphosate	81 / 78	1.00 (0.59–1.71)	0.94 (0.50–1.77)
Imazethapyr	30 / 41	1.10 (0.62–1.94)	1.09 (0.56–2.11)
Paraquat	31 / 24	1.11 (0.70–1.78)	1.01 (0.52–1.95)
Petroleum oil/distillates	44 / 46	1.13 (0.73–1.75)	1.36 (0.78–2.36)
**Fungicides/Fumigants**			
Benomyl	21 / *	1.52 (0.88–2.62)	1.75 (0.82–3.75)
Captan	13 / 11	1.29 (0.69–2.44)	0.72 (0.26–1.99)
Chlorothalonil	17 / *	1.49 (0.76–2.94)	0.85 (0.29–2.51)
Maneb	15 / *	0.85 (0.46–1.58)	1.44 (0.63–3.27)
Metalaxyl	38 / 23	1.42 (0.88–2.29)	1.05 (0.52–2.09)
Methylbromide	22 / 20	0.85 (0.49–1.48)	1.47 (0.68–3.19)

^1^Lifetime history ever used specific pesticides at enrollment (1993-1997) or first follow-up (1999-2003) prior to RA diagnosis.

^2^Among participants with ≥24 months continuous fee for service (FFS) coverage (Parts A and B coverage without Part C), including initial ≥12-month clean period with no claims for rheumatoid arthritis; specialist-confirmed cases had ≥2 claims for RA ≥30 days apart plus ≥1 claim(s) from a rheumatologist. Asterix (*) indicates percent not shown if number or percent not shown due to <11 in a cell or ability to infer the count of groups with <11 exposed cases in conjunction with other data presented.

^3^Relative Risk estimates calculated using log binomial regression, adjusted for categorical age (40-49, 50-59, 60-69, 70+), state (NC, IA), education (≤high school, >high school), and correlated pesticides (R2>0.35): [aldicarb (chlorothalonil and benomyl), chlorothalonil (aldicarb and benomyl), atrazine (alachlor), butylate (cyanazine and metribuzin)].

^4^Interaction p-values <0.20 for following pesticides by smoking (ever/never): aldrin (_int_p=0.18), carbofuran (_int_p =0.07), alachlor (_int_p=0.08), butylate (_int_p=0.19), atrazine (_int_p=0.14), chlorimuron ethyl (_int_p=0.13).

### Additional analyses

Given their frequent application together as product or tank-mixtures, we explored patterns of atrazine, alachlor and metolachlor use in relation to RA (Table 5). Both alachlor and metolachlor were frequently used by those who also used atrazine; 58% of non-cases used atrazine and either alachlor and/or metolachlor, 16% used only atrazine, and 8% used only alachlor and/or metalochlor, while 19% used none of these 3 herbicides. Altogether, compared to non-users, the risk of RA was elevated only among those who used atrazine as well as alachlor and/or metalochlor (1.84; 1.11–3.04).

**Table 5:** Risk of RA in relation to combined atrazine with alachlor and/or metolachlor use.

**Ever used**^**1**^:	Non-cases^2^	Specialist-confirmed cases^2^	Relative Risk^1^
	(*N* = 22,320)	(*N* = 161)	(95% CI)
	N (%)	N (%)	
Atrazine + alachlor and/or metolachlor			
None of these	3552 (18)	21 (15)	Reference
Alachlor and/or metolachlor, not atrazine	1534 (8)	< 11 (*)	1.39 (0.65–2.96)
Atrazine, not metolachlor or alachlor	3056 (16)	20 (15)	1.29 (0.70–2.39)
Atrazine, and alachlor and/or metolachlor	11,269 (58)	88 (63)	1.84 (1.11–3.04)

^1^Lifetime history ever used specific pesticides at enrollment (1993–1997) or first follow-up (1999–2003) prior to rheumatoid arthritis (RA) diagnosis.

^2^Among participants with ≥ 24 months continuous fee for service (FFS) coverage (Parts A and B coverage without Part C), including an initial ≥ 12-month clean period with no claims for rheumatoid arthritis; specialist-confirmed cases had ≥ 2 claims for RA ≥ 30 days apart plus claims from a rheumatologist. Asterix (*) indicates percent not shown if number or percent not shown due to < 11 in a cell or ability to infer the count of groups with < 11 exposed cases in conjunction with other data presented.

^3^Relative Risk estimates calculated using log binomial regression, adjusted for categorical age (40–49, 50–59, 60–69, 70+), state (NC, IA), education (≤ high school, > high school), and smoking pack year.

In ancillary analyses of overall cases, several associations with ever-use of specific pesticides were upheld (Supplementary Table 2), though the strength of effect estimates were somewhat attenuated, e.g., malathion (RR = 1.43; 95% CI 1.08–1.91), carbofuran (1.34; 1.04–1.72), metolachlor (1.32; 1.03–1.68), and benomyl (1.49; 1.08–2.06). In exposure-response models for cumulative IWLD (Supplementary Table 3), the trend for malathion was completely attenuated (RRs 0.76–1.04, p_trend_=0.70), while elevated RRs remained for lower or middle levels of carbofuran (M1 1.40; 1.00-1.97), alachlor (1.38;0.98–1.94), metolachlor (T1 1.56;1.11–2.20), and higher levels of butylate (M2 1.47;1.01–2.43). A new exposure-response was seen for turbufos [(M2 1.37;0.99–1.89) and (M1 1.12;0.77–1.61); p_trend_=0.06].

Finally, in a sensitivity analysis using a more stringent definition of incidence (i.e., ≥ 36 months complete continuous FFS coverage, including a clean period of 24 months; *N* = 14,916 total, 110 specialist confirmed cases), characteristics of incident RA cases were similar to the main analysis sample (Supplementary Table 4), except for lower median days mixed or applied pesticides among both overall and specialist-confirmed cases (though IQRs largely overlapped). In this smaller sample, we saw similar RR (though less precise CI) for specialist-confirmed RA and specific insecticides (Supplementary Table 5): malathion (RR 1.66;95%CI 1.03–2.70), phorate (1.48; 0.98–2.24), and carbaryl (1.51; 0.97–2.36), but decreases in magnitude (> 10%) for the two herbicides, alachlor (1.25; 0.85–1.84) and metolachlor (1.29; 0.87–1.91), while the RR for benomyl was increased by 21% (1.75; 1.05–2.90).

## Discussion

This study is the first to investigate use of specific pesticides and the development of specialist-confirmed RA using Medicare claims data linked to AHS participants. In analyses of data from licensed pesticide applicators, we report novel associations with several pesticides not seen in our prior work in male applicators based on self-reported RA questionnaire data with low follow-up response rates^14^. Our findings include dose-response associations with use of malathion and phorate, two commonly used organophosphate insecticides. We also report associations with two related carbamate insecticides, carbaryl and carbofuran, with a trend for greater IWLD use of carbofuran (but not carbaryl). Our current findings indicate a more complex relationship of RA and the herbicide atrazine than previously reported^14^, with no trend for overall atrazine use after adjusting for alachlor; but at the same time observed associations of RA with alachlor and metolachlor occurred largely among those who also used atrazine. Finally, we saw a new association of RA risk with the fungicide benomyl. Taken together, these results extend our prior findings in older AHS applicators.

Associations of RA with malathion and carbaryl are notable, as these are the two most commonly used insecticides in the cohort (reported by 73% and 62% of non-cases, and 81% and 75% of cases) and continue to be approved and are commonly used in the U.S. in agriculture and other settings. Although previous results did not indicate an overall association of self-reported RA with malathion, stratified analyses suggested an association (OR 1.55; 95%CI 0.89, 2.69) among those ages ≥ 50 years at AHS enrollment^14^. The observed exposure-response relationship showing greater risk of specialist-confirmed RA with increasing cumulative IWLD of malathion use supports these results, but this exposure-response was not observed when all cases were included (i.e., also including those with no RA specialist claims); this could suggest the association was specific to rheumatologist treated cases, maybe a more severe phenotype (or the more stringent case definition). The association of RA with ever use of carbaryl was consistent in various sensitivity and ancillary analyses, however we did not confirm prior suggestive findings of exposure-response in self-reported RA^14^, instead seeing the strongest association among those with lower IWLDs of use. Other evidence of immune- effects of these pesticides in AHS applicators includes an exposure-response association of malathion with shingles risk (including those without a prior RA, other SARD, or leukemia/lymphoma)^20^. Two highly toxic insecticides, phorate and carbofuran, were also associated with RA in the current study. Phorate could not be evaluated for exposure-response due to small numbers. Carbofuran has not been associated with self-reported RA, other SARD, or shingles in prior AHS investigations; however, in a small study of AHS applicators carbofuran was strongly associated with rare disease-specific extractable nuclear antibodies among those with anti-nuclear antibodies^14,20–22^.

We identified an association of RA with the fungicide benomyl. Although benomyl was not previously associated with self-reported RA^14^, it was associated with elevated shingles risk among AHS applicators without SARD or leukemia/lymphoma^20^. Though generally considered of low acute toxicity to mammals, benomyl is no longer used in the U.S. and other countries due to potential risks associated with chronic exposures^23^, possibly due to its metabolism to carbendazim, which remains widely used as a fungicide in global settings and associated with toxicity in animal models, including developmental, reproductive, endocrine, and hematological effects^24^. In prior analyses of female spouses, RA was associated with the fungicide maneb^15^. Both benomyl and maneb are carbamate fungicides, which is notable given associations in this study with other carbamate pesticides, such as carbaryl and carbofuran.

We observed associations of RA with two anilid/aniline herbicides, alachlor and metolachlor, however neither showed a clear exposure-response relationship. We did not confirm an independent association of atrazine with RA; previous results showed a significant trend for lifetime days of atrazine use and RA among male applicators, but these analyses did not adjust for exposure intensity weights or alachlor use^14^. In the current investigation, associations were most apparent among those using both atrazine and alachlor or metolachlor (commonly used together in product or tank mixtures)., This combined use was not evaluated in our prior analyses. Immune effects of alachlor in the AHS include an exposure-response association with self-reported shingles risk, and alachlor was among the top two pesticides involved in high pesticide exposure events associated with Medicare claims for shingles^20,25^. Another triazinone herbicide, metribuzin, was associated with RA, albeit at lower IWLD of use. Metribuzin is also used in combination with alachlor and metolachlor and results are consistent with suggestive findings for other immune endpoints in the AHS, including SARD and shingles^20,22^. Taken together, our results illustrate the importance of considering the combined effects of pesticides that are often used together or substituted over time. Further research is needed to understand risks associated with these herbicides, and the related herbicide acetochlor, approved for use by the Environmental Protection Agency in 1994 and first reported in later phases of AHS follow-up.

Smoking is the best-known environmental risk factor for RA; smoking-associated RA risk may be higher in older adults and males, those with genetic risk factors, and/or occupational exposure to silica and other inhaled substances in the development of RA^13,26–28^. We saw few differences in analyses stratified by history of cigarette smoking (ever versus never), nor evidence of effect modification of pesticide associations with RA by smoking except for two possible interactions (*p*< 0.10) with carbofuran and alachlor, both showing stronger associations in smokers. Cases who smoked also had a higher percentage who used carbofuran and alachlor, which could indicate an elevated risk profile. Some herbicide associations were also more apparent among smokers (e.g., butylate, atrazine, and another triazine herbicide, cyanazine), while other associations were similar or somewhat more apparent among non-smokers (e.g., malathion, carbaryl, metolachlor, benomyl). Although smoking increases RA risk, we did not hypothesize a particular direction of effect modification. Smoking could be the dominant risk factor in some individuals, perhaps a sufficient cause in the presence of certain genetic risk factors or other respiratory exposures, in which case pesticide associations could be more apparent among individuals developing RA through alternative causal pathways. Conversely, some pesticides may contribute to RA regardless of smoking, or perhaps increase susceptibility for immune dysregulation and autoimmunity together with smoking, genetic risk factors, or other occupational or respiratory exposures. Characterization of genetic risk factors and RA phenotype, and more precise data on the timing of exposures, are needed to better understand the role of pesticides, which might also be addressed by including pesticides in experimental studies of inflammatory arthritis^29–31^.The development of RA may begin years prior to clinical manifestations, with exposures impacting pre-clinical and clinical pathology at different stages in initiation of autoantibodies, epitope spreading, and inflammation^32,33^. The pesticides identified in the current study have a diverse range of potential non-cancer toxicities, with oral reference doses varying by orders of magnitude (ranging from 1 × 10 ^−1^ for carbaryl, to 5 × 10 ^−4^ for phorate, Supplementary Table 6). Many have neurotoxic effects and permanent or reversible acetylcholinesterase inhibition, and most have endocrine disrupting effects, though affected systems vary. Several pesticides associated with RA in this study may contribute to epigenetic changes and long-term effects on immunity relevant to RA^34–37^. Other pathways may include direct or indirect immunotoxicity resulting in immune dysregulation, inflammation, susceptibility to infections, or dysbiosis of the gut microbiota^38,39^. Malathion has clear evidence of immunosuppressive effects in experimental studies^40–42^; while exact mechanisms are not always apparent, both organophosphate and carbamate insecticides could manifest as neuroendocrine or immune effects through inhibition of the enzyme acetylcholinesterase^43^. Other indirect pathways may include impacts on synovitis through actions of carbamate insecticides on the melatonin receptor or other immune effects^44–46^. In addition to impacting neurotransmitters, various types of carbamate pesticides may exert direct and indirect effects on immunity by other pathways^44^. The lack of exposure-response observed for some pesticides could reflect a variety of scenarios, including low-dose effects (e.g., seen with some endocrine disrupting compounds), competing pathways, or the possibility that exposure intensity or timing relative to different stages of disease pathogenesis may be more important than duration or chronicity of use, or perhaps patterns of combined or substituted use of different pesticides over time. As these relationships are difficult to ascertain in observational human studies, experimental studies can help to address these possibilities. For example, combined atrazine and metolachlor exposure in an amphibian model led to greater thymic damage than either of the chemicals alone^47^. Further research is needed in experimental models of inflammatory arthritis and autoimmune pathologies.

Strengths of the AHS includes its large sample size of licensed pesticide applicators, prospective design, detailed pesticide data collected prior to RA diagnosis, and exposure-response data including an adjustment for intensity weights based on application methods and the use of personal protective equipment. Self-reported pesticide use in the AHS has been shown to be reliable^48^. Since exposure is impacted by application methods and use of protective equipment, an algorithm was applied to estimate cumulative IWLD of use, supported by measurement studies^49^. Complete data on lifetime ILWD at AHS enrollment was available on 22 pesticides (including carbofuran, atrazine, alachlor, metolachlor), while data on 28 others (including malathion, carbaryl, butylate, and metribuzin) were limited to applicators who completed the supplemental take-home questionnaire. The current analyses integrated data on additional pesticide use from the first follow-up survey (1999–2003), but ongoing and new pesticide use is plausible for many applicators who were still farming during follow-up and prior to RA diagnosis. Given ambiguous exposure timing relative to various stages of susceptibility and preclinical disease etiology, it is difficult to predict how misclassification could bias our findings. We limited examination of mixtures to the combination of atrazine with alachlor and/or metolachlor given prior findings of an association of RA with atrazine^14^. Results showed a stronger association for those who used atrazine together with alachlor and/or metolachlor, and further found that only 8% of non-cases used these herbicides but not atrazine, highlighting the potential influence of sparse data in models of atrazine adjusting for these correlated pesticides. Applicators routinely use more than one pesticide per crop and over time, and future studies may wish to examine other common mixtures and pesticides with similar modes of action, for example the insecticide malathion associated with RA for the first time in this analysis together the insecticide carbaryl, previously found to be associated with RA^14^. Farmers have higher occupational exposure to most pesticides, so these findings may not be generalized to the population. However, many pesticides associated with RA in this sample are currently approved for general residential or public health uses in the U.S. (e.g., malathion, carbaryl), and thus broadly impact the general population through direct (e.g., personal use) and indirect exposures (e.g., as food residues). Others, including atrazine and metolachlor may also be found in ground water, albeit at low concentrations^50,51^.

While some of the current findings are consistent with our prior work^14^, direct comparisons are limited by different study samples and case ascertainment methods. Previously cases were identified by self-reported diagnosis and DMARD use among those completing active follow-up and case confirmation surveys, and a small number with medical-records or validation by their physician. Age is an established risk factor for RA^3,4^, and the current study sample was older than the prior study sample. Besides being older at AHS enrollment and RA diagnosis, cases based on Medicare claims data in the current analysis were more likely to be from North Carolina (54% versus 43% in the prior study) and were less likely have an education beyond high school (22% vs. 38%) or to be current smokers (12% vs. 21%). Some pesticides were more prevalent in the current study, e.g., carbaryl was used by 75% of Medicare-based cases versus 57% of self-reported cases, which may reflect the inclusion of updated pesticide use data in the first follow-up and the older age of participants. This could yield different exposure distributions and observed exposure-response. Benomyl use, which was associated with RA in the current, and not the prior study, was also more common in Medicare-based cases (20% vs. 7% of self-reported cases). Very few applicators in the current study were female (not shown), while the prior study was limited to males. Altogether, a variety of factors may contribute to divergent results.

Use of Medicare claims data in the current study enabled more complete cohort follow-up and identification of cases based on visits for RA and specialist care. Claims-based algorithms restricting to 2 or more visits can accurately identify RA patients, with enhanced specificity for cases receiving specialist care^52,53^. We did not use DMARD-prescription data to further validate cases using Part D data, which covers only a fraction of cases starting in 2006; however, care by a rheumatologist is associated with DMARD use^54^. Our sample lacked those with Part A or Part C-only coverage, so participants could have changed coverage prior to RA diagnosis which might have influenced our findings if they were more or less likely to stay or become enrolled in FFS. In a sample limited to those with at least 36 months continuous FFS, using a stricter definition for incidence, results were not substantially changed. We performed ancillary analyses in overall cases, since some older patients in a rural environment may be less likely to access specialist care^19^; while most results did not markedly differ, some were attenuated. RA cases in the current study were almost entirely identified using ICD-9 codes, which provide no information on seropositive RA (e.g., based on rheumatoid factor (RF) or anti-citrullinated protein antibodies (ACPA)), only recently distinguished in by ICD-10 codes available in Medicare starting October 2015. Given strong associations and interaction of smoking with genetic factors and RF or ACPA-positive RA^26^, smoking stratified models may reflect possible phenotypic differences.

Compared to baseline models adjusting for age, state, and correlated pesticides, we saw limited evidence of additional confounding by education and smoking. However, residual confounding may occur due to time-varying or passive smoking exposures, for example in childhood. RA risk has also been associated with infections, diet, alcohol use, and body mass index^3,4^. The latter two factors were not included in final models due to no association with RA in exploratory baseline models, while the former were not evaluated in the current study. Infections linked to RA could exist on the causal pathway, to the extent that the immune response may be modified by acute or chronic pesticides exposures^20,25^. Farmers experience other immune modifying exposures, such as inorganic and organic dusts, solvents, and UV exposure. Our prior research has shown associations of self-reported RA with applying fertilizers, solvents, or painting^55^. Other studies suggest a role for diverse inhaled exposures in risk of RA^6,13,56^. Future studies should explore the interaction of pesticides with associated exposures, e.g., work with animals, and exposures to organic and inorganic (e.g., soil) dusts and potential contaminants^57^.

In sum, our findings support the hypothesis that specific pesticides may contribute to RA risk. In the AHS-Medicare linked cohort, several pesticides, including some currently approved and commonly used in agricultural, public health, or residential settingsmay increase RA risk among older adults. Replication of these findings in other populations and experimental studies is needed, given the potential implications for other autoimmune diseases and millions of tons of pesticide active ingredients produced and used in the U.S. and globally.

## Methods

### Population and sample

The study sample was drawn from the Agricultural Health Study (AHS), a prospective cohort of ~ 89,000 licensed pesticide applicators and spouses enrolled from 1993 to 1997 in North Carolina (NC) and Iowa (IA). Details of the enrollment protocol and population characteristics have been previously described^58^. Applicators enrolled in the cohort when they completed a questionnaire at the time they sought or renewed their pesticide application license (80% response). Enrolled applicators (*N* = 52,394 private applicators) were given a supplemental questionnaire to complete at home (44% response). Data were collected on demographics, pesticide use, and medical history (http://aghealth.nih.gov/collaboration/questionnaires.html). The current study also updated pesticide use information from the first follow-up survey 1999–2003 (64% response) for those with data on new or ongoing use prior to any RA claims. The study was approved by the institutional review boards of participating institutes (the National Cancer Institution and National Institution of Environmental Health Sciences and is currently approved under continuing review by the National Institutes of Health Institutional Review Board FWA000058, IRB IDOH93NCN013 / MODCR001011). The linkage to claims data was also approved by the Centers for Medicare and Medicaid Services (RSCH 2018–52174, DUA 52174). All methods were performed in accordance with the relevant guidelines and regulations. Informed consent was obtained from all participants following procedures approved by the Institutional Review Board at enrollment and follow-up.

Linkage of the AHS to Medicare administrative healthcare data was completed for 28,057 private applicators, 98% of the Medicare- eligible participants ages ≥ 65 years between 1999 and 2016^17^. Sample derivation for analyses of incident RA is shown in Supplementary Fig. 1. After excluding 5,654 individuals [(20%; 5,415 with < 24 months continuous FFS coverage (i.e., Medicare Parts A and B without Part C managed care insurance), 200 with prevalent RA and 39 with equivocal RA], the eligible study sample included 22,403 applicators. Of these, 319 were identified overall as incident RA cases, including 161 who were confirmed by specialist claims and were the focus of our primary analysis (described below). For statistical modeling, the analysis also excluded 1,968 participants with missing covariates (as listed below; 20,435 remaining).

### Case ascertainment

Claims for RA diagnosis included ICD9 7140, 7141, 7142, 71,481 or ICD10 M069, M0500, M0530, M0560, and M0510. Incident cases were identified following ≥ 12 months continuous FFS with no claims for RA and ≥ 12 additional months of continuous FFS. Both prevalent and incident cases were identified based on having ≥ 2 claims for RA ≥ 30 days apart; among these, we enumerated a subset of cases based on having at least one specialist claim for RA (CMS Specialist code 66, i.e., “specialist-confirmed cases”) to increase specificity of this claims-based algorithm^52,53^. Prevalent cases were identified if they had ≥ 2 claims for RA ≥ 30 days apart during the first 12 months of FFS coverage, while equivocal cases were identified if they had 1 claim for RA (or 2 within 30 days) in the first 12 months, but no additional claims for RA during follow-up.

### Pesticide exposure and covariate data

At enrollment applicators were asked about ever-use of 50 pesticides; for 22 of these, further questions asked about categorical frequency (days per year) and duration (years) of use. A supplemental questionnaire asked about duration and frequency for the remaining 28 pesticides. Updated use of all 50 pesticides in the first follow-up survey (1999–2003) was incorporated into exposure data for those completing the survey and who had follow-up time (as cases or non-cases) after the age at completion. Intensity-weighted lifetime days of use (IWLD) was calculated using category midpoints for years and days per year, multiplied by an intensity score incorporating data on factors impacting personal exposures (e.g., personal protective equipment and different application methods)^49^. We derived categories of IWLD for exploration of exposure response (median split, tertiles, and quartiles) versus never-use based on the percent exposed cases (i.e., median split for 30 < 50%, tertiles for 50 < 70%, and quartiles for ≥ 70% exposed). We did not evaluate exposure-response if the prevalence of use was < 30%.

Covariates included in primary analyses are age at enrollment (40–49, 50–59, 60–69, 70+), state (Iowa vs. North Carolina), education (high school or less vs. higher education), and smoking pack-years, and correlated pesticides as described below. Those missing data on state, education, or smoking were excluded from modeling. We included total lifetime days mixed or applied pesticides in Table 5 to provide readers a sense of the distribution of general pesticide use, but they were not considered as potential confounders. Body mass index and current alcohol use showed no association with RA in exploratory models, so were not included as covariates.

### Analyses

Our analyses focused on history of ever using of 44 specific pesticide active ingredients with ≥ 11 exposed specialist-confirmed RA cases. We conducted log binomial regression to calculate relative risks (RR) and 95% Confidence Intervals (CI). Models adjusted for age, state, education, and smoking pack-years; for a subset of pesticides, we adjusted for any correlated pesticides with R2 > 0.35 and an effect estimate obtained prior to co-exposure adjustment was 1.20 or greater. Analyses used data releases P1REL201701.00, P2REL201701.00, P3REL201809.00, AHSREL201706.00, and were conducted using SAS version 9.4 (SAS Institute, Cary, North Carolina).

Exposure response was assessed through visual examination of stepwise increasing RR across increasing categories of IWLD for pesticides (described above). Trend tests used an ordinal variable based on the midpoint of each IWLD category, with never-use as the lowest category. We also explored potential differences in specific pesticide associations (ever use) with RA stratified by smoking history (ever vs. never). Effect measure modification was assessed for descriptive purposes by inclusion of an interaction term of smoking by pesticide use.

We conducted several additional analyses. First, we considered combined herbicide use for atrazine and alachlor or metolachlor, given their frequent use together. In ancillary analyses, we also examined associations in the larger sample of cases (i.e., overall incident cases) regardless of specialist claims. Finally, we conducted an analysis that required continuous FFS coverage (without gaps in available claims data due to lack of insurance or having periods of Part A-only (hospitalization) or Part C/managed care insurance), as well as ≥ 36 months of FFS coverage, following a 24-month clean period, as cases in remission may receive less regular care.

## Supplementary Information

Supplementary Material 1.

## Data Availability

The datasets used during these analyses are not publicly available due to policies protecting administrative healthcare claims data from the Centers for Medicare and Medicaid Services (CMS) linked to the Agricultural Health Study (AHS) cohort. Requests to analyze these data can be directed to the corresponding author, and may be granted following study policies, including AHS Executive Committee review (https://aghealth.nih.gov/collaboration/process.html) and a data use agreement approved by the Centers for Medicare and Medicaid Services.
