# Supplementary material for: Associations between pesticide use and rheumatoid arthritis among older farmers in the Agricultural Health Study
Source: Sci Rep. 2024 Dec 2;14:29978. doi: 10.1038/s41598-024-76179-2 (PMC11611918; doi:10.1038/s41598-024-76179-2)
Supplement: Supplementary file 1 — Supplementary Material 1. [file 41598_2024_76179_MOESM1_ESM.docx]

**Supplementary Materials:** Associations between Pesticide Use and Rheumatoid Arthritis in the Agricultural Health Study Medicare-linked Cohort

Christine G. Parks^1^, Darya Leyzarovich^2^, Ghassan B. Hamra^2^, Karen H. Costenbader^3^, Dazhe Chen^1^, Jonathan N. Hofmann^4^, Laura E. Beane Freeman^4^, Dale P. Sandler^1^

^1^Epidemiology Branch, National Institute of Environmental Health Sciences, National Institutes of Health, Research Triangle Park, North Carolina, U.S.A.; ^2^DHL, LLC, Bethesda, Maryland, U.S.A.; ^3^Brigham and Women's Hospital, Harvard Medical School, Boston, Massachusetts, USA.; ^4^Occupational and Environmental Epidemiology Branch, Division of Cancer Epidemiology and Genetics, National Cancer Institute, Bethesda, Maryland, U.S.A.

**Includes 1 Figure and 5 Tables:**

Supplemental Figure 1. Sample Derivation (PP. 2-3)

Supplementary Table 1. List of correlated pesticides (P. 4)

Supplementary Table 2. Ever use of specific pesticides and RA: overall cases (PP.5-6)

Supplementary Table 3. Intensity weighted lifetime days used pesticides and RA: overall cases (PP.7-9)

Supplementary Table 4. Characteristics of AHS applicators with ≥36 months FFS (P. 10)

Supplemental Table 5. Ever use of specific pesticides and RA risk among AHS applicators with ≥36 FFS: specialist-confirmed cases (PP. 11-12).

Supplemental Table 6. Non-cancer reference dose (RfD) for chronic oral exposure and human health systems affected: specific pesticides associated with RA risk among AHS applicators with ≥24 months continuous Medicare-FFS (1999-2016).





Supplementary Figure 1. Study population-exclusions-and analytic sample derivation. Prevalent and incident cases are those with ≥2 or more claims ≥30 days apart. Prevalent cases had their first claim for RA recorded in the first 12 months of continuous fee for service (FFS) coverage (Parts A and B without Part C). Equivocal cases are those with 1 claim or 2 claims less than 30 days apart in the first 12 months-with no additional claims for RA during follow-up. Incident cases were participants with ≥24 months continuous FFS coverage (Parts A and B, without Part C), including an initial ≥12-month clean period with no claims for rheumatoid arthritis. Additionally, specialist confirmed cases required ≥1 claim for RA from a rheumatologist. In sensitivity analyses, we restricted the sample to those with ≥36 months continuous fee for service (FFS) coverage, including an additional ≥12-month clean period (i.e., 13-24 months) with no RA claims in which cases could be identified as prevalent or equivocal. Incident and specialist-confirmed cases were classified as having an initial 24 months without an RA claim.

Supplementary Table 1. List of correlated pesticides (r2>0.35) for pesticides with RR>1.2^1^

|  | Evaluated for confounding |
| --- | --- |
| Model |  |
|  |  |
| A | Toxaphene |
|  | Parathion |
|  |  |
| B | Malathion |
|  | Carbofuran |
| C | Aldicarb |
|  | Benomyl |
|  | Chlorothalonil |
|  |  |
| D | Alachlor |
|  | Atrazine |
|  |  |
| E | Trifluralin |
|  | Butylate |
|  |  |
| F | Cyanazine |
|  | Atrazine |
|  |  |
| G | Metribuzin |
|  | Cyanazine |
|  | Butylate |
|  |  |
| H | Benomyl |
|  | Chlorothalonil |
|  |  |

^1^ Used for covariate adjustment to address potential confounding.

Supplementary Table 2. Ever use of specific pesticides and RA risk among AHS applicators with ≥24 months continuous Medicare-FFS (1999-2016): overall cases

|  | | **Non-cases^2^** | | **Overall cases^2^** | | **Multivariable adjusted** | | |
| --- | --- | --- | --- | --- | --- | --- | --- | --- |
|  | | **(N = 22,084)** | | **(N=319)** | | **Relative Risk^3^** | | |
| **Ever used^1^:** | | N (%) | | N (%) | | (95% Confidence Interval) | | |
| **Insecticides** | |  | |  | |  | | |
| Organochlorines | |  | |  | |  | | |
| Aldrin | | 6654 (33) | | 86 (31) | | 0.95 (0.73-1.24) | | |
| Chlordane | | 7273 (36) | | 100 (36) | | 0.93 (0.73-1.20) | | |
| DDT | | 9204 (45) | | 144 (51) | | 1.02 (0.81-1.30) | | |
| Heptachlor | | 5271 (26) | | 69 (25) | | 1.05 (0.78-1.41) | | |
| Lindane | | 4785 (24) | | 69 (25) | | 1.23 (0.93-1.62) | | |
| Toxaphene | | 4332 (21) | | 73 (26) | | 1.18 (0.90-1.55) | | |
| Organophosphates | |  | |  | |  | | |
| Chlorpyrifos | | 8429 (41) | | 108 (38) | | 1.00 (0.79-1.27) | | |
| Coumaphos | | 1714 (9) | | 22 (8) | | 1.07 (0.69-1.65) | | |
| Diazinon | | 7267 (36) | | 101 (36) | | 1.00 (0.78-1.29) | | |
| Dichlorvos | | 2102 (10) | | 22 (8) | | 0.96 (0.62-1.49) | | |
| Fonofos | | 4412 (22) | | 46 (17) | | 0.91 (0.65-1.28) | | |
| Malathion | | 14913 (73) | | 219 (78) | | 1.43 (1.08-1.91) | | |
| Parathion | | 3832 (19) | | 64 (23) | | 1.12 (0.84-1.49) | | |
| Phorate | | 7614 (38) | | 100 (36) | | 1.20 (0.92-1.58) | | |
| Terbufos | | 7308 (36) | | 99 (36) | | 1.26 (0.97-1.64) | | |
| Carbamates | |  | |  | |  | | |
| Aldicarb | | 2097 (10) | | 38 (14) | | 1.07 (0.72-1.58) | | |
| Carbaryl | | 12679 (62) | | 195 (69) | | 1.22 (0.94-1.60) | | |
| Carbofuran | | 6105 (30) | | 94 (35) | | 1.34 (1.04-1.72) | | |
| Pyrethroids | |  | |  | |  | | |
| Permethrin_C | | 2246 (11) | | 30 (11) | | 1.09 (0.74-1.59) | | |
| **Herbicides** | |  | |  | |  | | |
| Analids/analines | |  | |  | |  | | |
| Alachlor | | 10815 (54) | | 152 (55) | | 1.24 (0.97-1.58) | | |
| Metolachlor | | 8877 (44) | | 125 (46) | | 1.32 (1.03-1.68) | | |
| Pendimethalin | | 8869 (44) | | 103 (37) | | 0.85 (0.66-1.08) | | |
| Trifluralin | | 10350 (51) | | 125 (46) | | 1.01 (0.78-1.31) | | |
| Phenoxy | |  | |  | |  | | |
| 2_4_D | | 16325 (79) | | 212 (74) | | 0.93 (0.71-1.23) | | |
| 2_4_5_T | | 6503 (32) | | 83 (30) | | 0.91 (0.70-1.18) | | |
| 2,4,5-TP | | 2587 (13) | | 30 (11) | | 0.87 (0.59-1.27) | | |
| Thiocarbamate |  | |  | |  | |  |  |
| Butylate | | 6613 (33) | | 87 (32) | | 1.15 (0.86-1.52) | | |
| Triazine/Triazinone | |  | |  | |  | | |
| Atrazine | | 14822 (74) | | 197 (72) | | 1.02 (0.76-1.36) | | |
| Cyanazine | | 8072 (40) | | 99 (36) | | 1.13 (0.86-1.49) | | |
| Metribuzin | | 9163 (45) | | 116 (42) | | 1.17 (0.90-1.51) | | |
| Other | |  | |  | |  | | |
| Chlorimuron Ethyl | | 6800 (34) | | 85 (31) | | 1.03 (0.79-1.33) | | |
| Dicamba | | 9797 (49) | | 107 (39) | | 0.92 (0.69-1.22) | | |
| Glyphosate | | 16476 (80) | | 225 (79) | | 0.98 (0.74-1.31) | | |
| Imazethapyr | | 7629 (38) | | 73 (27) | | 0.78 (0.57-1.07) | | |
| Paraquat | | 4852 (24) | | 78 (28) | | 1.11 (0.84-1.46) | | |
| Petrolium_Oil | | 9130 (45) | | 108 (39) | | 0.91 (0.71-1.18) | | |
| **Fungicides and Fumimgants** | |  | |  | |  | | |
| Benomyl | | 2390 (12) | | 53 (19) | | 1.49 (1.08-2.06) | | |
| Captan | | 2099 (10) | | 35 (13) | | 1.32 (0.93-1.89) | | |
| Chlorothalonil | | 1644 (8) | | 31 (11) | | 0.90 (0.58-1.40) | | |
| Maneb | | 2325 (12) | | 39 (14) | | 0.98 (0.69-1.40) | | |
| Metalaxyl | | 4760 (23) | | 85 (30) | | 1.20 (0.90-1.58) | | |
| Methylbromide | | 3383 (16) | | 64 (22) | | 1.09 (0.80-1.49) | | |

FFS = Fee for Service; DDT-Dichlorodiphenyltrichloroethane; EPTC-S-Ethyl dipropylthiocarbamate.

**^1^**Lifetime history ever used specific pesticides at enrollment (1993-1997) or first follow-up (1999-2003) prior to rheumatoid arthritis (RA) diagnosis.

**^2^**Among participants with ≥24 months continuous fee for service (FFS) coverage (≥11 months per year Parts A and B coverage without Part C) including an initial ≥12-month clean period with no claims for rheumatoid arthritis; cases had 2+ claims for RA ≥30 days apart, regardless of specialist claims.

**^3^**Relative Risk estimates calculated using log binomial regression-adjusted for categorical age at enrollment (40-49, 50-59, 60-69, 70+), state (NC-IA), education (≤high school->high school), smoking pack years, and correlated pesticides (R2>0.35): aldicarb (chlorothalonil and benomyl)-chlorothalonil (aldicarb and benomyl)-atrazine (alachlor)-butylate (cyanazine and metribuzin).

Supplementary Table 3. Intensity weighted lifetime days used pesticides and RA risk among AHS applicators with ≥24 months continuous FFS Medicare coverage (1999-2016): overall cases

|  | **Non-cases^2^** | | **Overall cases^2^** | | | **Relative Risk^3^** | | |  |  |
| --- | --- | --- | --- | --- | --- | --- | --- | --- | --- | --- |
| **IWLD categories^1^** | (N=22,084) | | (N=319) | | | (95% CI) | | |  |  |
|  | n (%) | | n (%) | | |  | | |  |  |
| **Insecticides** |  | |  | | |  | | |  |  |
| Organochlorine |  | |  | | |  | | |  |  |
| DDT* |  | |  | | |  | | |  |  |
| None | 6320 (63) | | 93 (60) | | | reference | | |  |  |
| M1 | 1855 (19) | | 33 (21) | | | 1.02 (0.69-1.53) | | |  |  |
| M2 | 1849 (18) | | 29 (19) | | | 0.81 (0.53-1.24) | | |  |  |
| *p-trend* |  | |  | | | 0.40 | | |  |  |
| Organophosphate |  | |  | | |  | | |  |  |
| Chlorpyrifos |  | |  | | |  | | |  |  |
| None | 10651 (56) | | 148 (58) | | | reference | | |  |  |
| M1 | 4103 (22) | | 51 (20) | | | 0.98 (0.72-1.35) | | |  |  |
| M2 | 4101 (22) | | 56 (22) | | | 1.08 (0.80-1.47) | | |  |  |
| *p-trend* |  | |  | | | 0.65 | | |  |  |
| Malathion* |  | |  | | |  | | |  |  |
| Never | 3297 (31) | | 54 (35) | | | reference | | |  |  |
| T1 | 2476 (23) | | 26 (17) | | | 0.76 (0.48-1.22) | | |  |  |
| T2 | 2424 (23) | | 35 (22) | | | 1.01 (0.66-1.55) | | |  |  |
| T3 | 2437 (23) | | 41 (26) | | | 1.04 (0.69-1.56) | | |  |  |
| p-trend |  | |  | | | 0.70 | | |  |  |
| Turbufos |  | |  | | |  | | |  |  |
| None | 11293 (62) | | 145 (61) | | | reference | | |  |  |
| M1 | 3528 (19) | | 40 (17) | | | 1.12 (0.78-1.61) | | |  |  |
| M2 | 3518 (19) | | 51 (22) | | | 1.37 (0.99-1.89) | | |  |  |
| *p-trend* |  | |  | | | 0.06 | | |  |  |
| Carbamates |  | |  | | |  | | |  |  |
| Carbaryl* |  | |  | | |  | | |  |  |
| None | 5205 (49) | | 67 (41) | | | reference | | |  |  |
| T1 | 1803 (17) | | 31 (19) | | | 1.35 (0.88-2.06) | | |  |  |
| T2 | 1810 (17) | | 35 (21) | | | 1.33 (0.86-2.05) | | |  |  |
| T3 | 1802 (17) | | 32 (19) | | | 1.06 (0.65-1.71) | | |  |  |
| *p-trend* |  | |  | | | 0.56 | | |  |  |
| Carbofuran |  | |  | | |  | | |  |  |
| None | 12363 (68) | | 149 (63) | | | reference | | |  |  |
| M1 | 2921 (16) | | 43 (18) | | | 1.40 (1.00-1.97) | | |  |  |
| M2 | 2909 (16) | | 46 (19) | | | 1.33 (0.96-1.84) | | |  |  |
| *p-trend* |  | |  | | | 0.04 | | |  |  |
| **Herbicides** |  | |  | | |  | | |  |  |
| Anilids/anilines |  | |  | | |  | | |  |  |
| Alachlor |  | |  | | |  | | |  |  |
| None | 7999 (44) | | 98 (41) | | | reference | | |  |  |
| T1 | 3425 (19) | | 44 (19) | | | 1.23 (0.86-1.77) | | |  |  |
| T2 | 3430 (19) | | 51 (22) | | | 1.38 (0.98-1.94) | | |  |  |
| T3 | 3433 (19) | | 44 (19) | | | 1.12 (0.78-1.59) | | |  |  |
| *p-trend* |  | |  | | | 0.26 | | |  |  |
| Metolachlor |  | |  | | |  | | |  |  |
| None | 9855 (54) | | 124 (51) | | | reference | | |  |  |
| T1 | 2792 (15) | | 46 (19) | | | 1.56 (1.11-2.20) | | |  |  |
| T2 | 2829 (15) | | 32 (13) | | | 1.11 (0.75-1.64) | | |  |  |
| T3 | 2805 (15) | | 40 (17) | | | 1.33 (0.93-1.90) | | |  |  |
| *p-trend* |  | |  | | | 0.12 | | |  |  |
| Pendimethalin* |  | |  | | |  | | |  |  |
| None | 6461 (61) | | 112 (70) | | | reference | | |  |  |
| M1 | 2048 (19) | | 23 (14) | | | 0.73 (0.47-1.15) | | |  |  |
| M2 | 2046 (19) | | NS (*) | | | 0.77 (0.50-1.18) | | |  |  |
| *p-trend* |  | |  | | | 0.13 | | |  |  |
| Trifluralin |  | |  | | |  | | |  |  |
| None | 8343 (46) | | 124 (52) | | | reference | | |  |  |
| T1 | 3304 (18) | | 41 (17) | | | 1.02 (0.71-1.46) | | |  |  |
| T2 | 3314 (18) | | 34 (14) | | | 0.89 (0.60-1.32) | | |  |  |
| T3 | 3300 (18) | | 41 (17) | | | 1.06 (0.74-1.53) | | |  |  |
| *p-trend* |  | |  | | | 0.95 | | |  |  |
| Phenoxy |  | |  | | |  | | |  |  |
| 2,4-D |  | |  | | |  | | |  |  |
| None | 4225 (21) | | 73 (26) | | | reference | | |  |  |
| Q1 | 3962 (20) | | 55 (20) | | | 0.90 (0.63-1.28) | | |  |  |
| Q2 | 3989 (20) | | 46 (17) | | | 0.82 (0.56-1.19) | | |  |  |
| Q3 | 3972 (20) | | 50 (18) | | | 0.88 (0.61-1.29) | | |  |  |
| Q4 | 3976 (20) | | 53 (19) | | | 0.92 (0.64-1.33) | | |  |  |
| *p-trend* |  | |  | | | 0.64 | | |  |  |
| Thiocarbamate |  | |  | | |  | | |  |  |
| Butylate* |  | |  | | |  | | |  |  |
| None | 7267 (73) | | 111 (*) | | | reference | | |  |  |
| M1 | 1362 (14) | | NS (*) | | | 0.56 (0.29-1.09) | | |  |  |
| M2 | 1343 (13) | | 28 (*) | | | 1.57 (1.01-2.43) | | |  |  |
| *p-trend* |  | |  | | | 0.16 | | |  |  |
| Triazine/Triazinone |  |  | |  |  | |  |  | |  |
| Atrazine |  | |  | | |  | | |  |  |
| None | 5228 (27) | | 81 (30) | | | reference | | |  |  |
| Q1 | 3600 (18) | | 50 (19) | | | 1.01 (0.70-1.46) | | |  |  |
| Q2 | 3604 (18) | | 46 (17) | | | 0.93 (0.63-1.36) | | |  |  |
| Q3 | 3607 (18) | | 45 (17) | | | 0.91 (0.61-1.34) | | |  |  |
| Q4 | 3605 (18) | | 46 (17) | | | 0.89 (0.60-1.31) | | |  |  |
| *p-trend* |  | |  | | | 0.46 | | |  |  |
| Cyanazine |  | |  | | |  | | |  |  |
| None | 10586 (58) | | 147 (61) | | | reference | | |  |  |
| M1 | 3900 (21) | | 49 (20) | | | 1.20 (0.85-1.71) | | |  |  |
| M2 | 3901 (21) | | 46 (19) | | | 1.13 (0.80-1.62) | | |  |  |
| *p-trend* |  | |  | | | 0.40 | | |  |  |
| Metribuzin* |  | |  | | |  | | |  |  |
| None | 6268 (61) | | 102 (65) | | | reference | | |  |  |
| M1 | 1958 (19) | | 34 (22) | | | 1.40 (0.92-2.11) | | |  |  |
| M2 | 1986 (19) | | NS (*) | | | 0.85 (0.53-1.38) | | |  |  |
| *p-trend* |  | |  | | | 0.85 | | |  |  |
| Other |  | |  | | |  | | |  |  |
| Dicamba |  | |  | | |  | | |  |  |
| None | 8829 (48) | | 140 (57) | | | reference | | |  |  |
| T1 | 3136 (17) | | 32 (13) | | | 0.89 (0.58-1.34) | | |  |  |
| T2 | 3128 (17) | | 40 (16) | | | 1.12 (0.76-1.65) | | |  |  |
| T3 | 3126 (17) | | 32 (13) | | | 0.90 (0.60-1.37) | | |  |  |
| *p-trend* |  | |  | | | 0.89 | | |  |  |
| Glyphosate |  | |  | | |  | | |  |  |
| None | 4174 (21) | | 66 (23) | | | reference | | |  |  |
| Q1 | 3963 (20) | | 57 (20) | | | 0.94 (0.66-1.33) | | |  |  |
| Q2 | 3984 (20) | | 50 (18) | | | 0.82 (0.57-1.19) | | |  |  |
| Q3 | 3961 (20) | | 50 (18) | | | 0.85 (0.59-1.23) | | |  |  |
| Q4 | 3961 (20) | | 60 (21) | | | 0.98 (0.69-1.41) | | |  |  |
| *p-trend* |  | |  | | | 0.75 | | |  |  |
| Imazethapyr |  | |  | | |  | | |  |  |
| None | 10811 (60) | | 167 (71) | | | reference | | |  |  |
| M1 | 3645 (20) | | 37 (16) | | | 0.85 (0.58-1.26) | | |  |  |
| M2 | 3648 (20) | | 32 (14) | | | 0.77 (0.51-1.15) | | |  |  |
| *p-trend* |  | |  | | | 0.18 | | |  |  |

IWLD-Intensity-weighted lifetime days used; NS or (*)-number or percent not shown due to <11 in a cell or ability to infer the count of groups with <11 exposed cases.

**^1^** As reported in 1993-97 and updated 1999-2003. Scores grouped based on percent exposed cases: median (30<50%)-tertiles (50<70%)-or quartiles (≥70%) as intensity-weighted lifetime days (IWLD) use of specific pesticides up through enrollment (1993-1997) or first follow-up (1999-2003) prior to RA diagnosis; *Indicates pesticides with IWLD data based only on the take home questionnaire.

**^2^**Among participants with ≥24 months continuous fee for service (FFS) coverage (Parts A and B coverage without Part C) including an initial ≥12-month clean period with no claims for rheumatoid arthritis; specialist-confirmed cases had 2+ claims for RA ≥30 days apart plus claims from a rheumatologist.

**^3^**Relative Risk estimates calculated using log binomial regression-adjusted for categorical age at enrollment (40-49, 50-59, 60-69, 70+), state, (NC-IA)-education (≤high school->high school), smoking pack years, and correlated pesticides (R2>0.350) [aldicarb (chlorothalonil and benomyl)-chlorothalonil (aldicarb and benomyl)-atrazine (alachlor)-butylate (cyanazine and metribuzin)].

Supplementary Table 4. Characteristics of AHS applicators with ≥36 months complete continuous Medicare-FFS (1999-2016): non-cases, overall and specialist-confirmed incident RA cases

|  |  | **Non-cases^2^** | **Overall Cases** | **Specialist-confirmed** |
| --- | --- | --- | --- | --- |
| Enrollment and patient factors^1^ |  | N=14,699 | N=217 | N=110 |
| Continuous FFS years (median-IQR) |  | 8.9 (4.8, 13.5) | 4.6 (2.0, 8.4) | 4.8 (1.8-8.6) |
| Diagnosis age (median-IQR) |  | --- | 74.4 (70.5, 77.9) | 73.5 (69.5, 77.2) |
| **Enrollment** |  |  |  |  |
| Age years (median-IQR) |  | 58 (52, 64) | 60 (57, 66) | 60 (55, 64) |
| State (n, %) |  |  |  |  |
| Iowa |  | 9414 (64.0%) | 112 (51.6%) | 57 (51.8%) |
| North Carolina |  | 5285 (36.0%) | 105 (48.4%) | 53 (48.2%) |
| Education (n, %) |  |  |  |  |
| ≤High school |  | 9964 (67.8%) | 171 (78.8%) | 84 (76.4%) |
| >High school |  | 4735 (32.2%) | 46 (21.2%) | 26 (23.6%) |
| Smoking (n, %)  Ever |  | 7844 (53.4%) | 126 (58.1%) | 65 (59.1%) |
| Past  Current |  | 6110 (41.6%)  1734 (11.8%) | 105 (48.4%)  21 (9.7%) | 53 (48.2%)  12 (10.9%) |
| Pack-years (median-IQR) |  | 0.5 (0.0, 15.0) | 2.3 (0.0, 18.8) | 2.4 (0.0, 15.0) |
| Lifetime days mixed/applied pesticides (median-IQR) |  | 225 (88, 508) | 179 (64, 482) | 179 (64, 457) |

FFS = Fee for Service; IQR = cut-points for first and 3^rd^ quartiles; NS=not shown due to <11 cases in

^1^Sample includes about 97% white males. Thus-the number of female and non-white cases-non-smokers-and missing smoking data are not shown-due to Ns <11 or derivatives that may reveal number <11 cases. More than high school Includes at least some college, or associates-or 4-year college degree.

**^2^**Among participants with 36 months continuous fee for service (FFS) coverage (Parts A and B coverage without Part C) including an initial ≥24-month clean period with no claims for rheumatoid arthritis; incident cases had 2+ claims for RA ≥30 days apart-while specialist-confirmed cases also included claims for RA from a rheumatologist.

Supplementary Table 5. Ever use of specific pesticides and RA risk among AHS applicators with ≥36 months continuous Medicare-FFS (1999-2016): specialist-confirmed cases.

|  | **Non-cases^2^** | | | **Cases^2^** | |  | | |
| --- | --- | --- | --- | --- | --- | --- | --- | --- |
|  | (N = 14,699) | | | (N=110) | | **Relative Risk^3^** | | |
| **Ever used^1^:** | N (%) | | | N (%) | | (95% Confidence Interval) | | |
| **Insecticides** |  | | |  | |  | | |
| Organochlorines |  | | |  | |  | | |
| Aldrin | 5178 (35) | | | 35 (32) | | 0.95 (0.62-1.45) | | |
| Chlordane | 5359 (36) | | | 36 (33) | | 0.86 (0.57-1.29) | | |
| DDT | 6939 (47) | | | 53 (49) | | 0.95 (0.64-1.39) | | |
| Heptachlor | 4043 (27) | | | 24 (22) | | 0.85 (0.52-1.39) | | |
| Lindane | 3525 (24) | | | 27 (25) | | 1.22 (0.78-1.91) | | |
| Toxaphene | 3351 (23) | | | 32 (29) | | 1.29 (0.85-1.97) | | |
| Organophosphates |  | | |  | |  | | |
| Chlorpyrifos | 6160 (41) | | | 44 (41) | | 1.07 (0.73-1.57) | | |
| Diazinon | 5243 (36) | | | 38 (35) | | 0.94 (0.63-1.41) | | |
| Dichlorvos | 1526 (10) | | | 11 (10) | | 1.22 (0.64-2.30) | | |
| Fonofos | 3255 (22) | | | 22 (21) | | 1.16 (0.70-1.91) | | |
| Malathion | 11033 (74) | | | 88 (81) | | 1.66 (1.03-2.70) | | |
| Parathion | 2811 (19) | | | 23 (21) | | 1.00 (0.62-1.61) | | |
| Phorate | 5738 (39) | | | 46 (42) | | 1.48 (0.98-2.24) | | |
| Terbufos | 5515 (38) | | | 43 (41) | | 1.40 (0.94-2.11) | | |
| Carbamates |  | | |  | |  | | |
| Aldicarb | 1558 (11) | | | 16 (15) | | 0.97 (0.52-1.80) | | |
| Carbaryl | 9183 (62) | | | 79 (72) | | 1.51 (0.97-2.36) | | |
| Carbofuran | 4511 (31) | | | 37 (36) | | 1.35 (0.90-2.02) | | |
| Pyrethroids |  | | |  | |  | | |
| Permethrin_C | 1640 (11) | | | 11 (10) | | 0.98 (0.53-1.84) | | |
| **Herbicides** |  | | |  | |  | | |
| Analids/analines |  | | |  | |  | | |
| Alachlor | 8100 (55) | | | 62 (58) | | 1.25 (0.85-1.84) | | |
| Metolachlor | 6707 (46) | | | 51 (48) | | 1.29 (0.87-1.91) | | |
| Pendimethalin | 6610 (45) | | | 47 (43) | | 0.99 (0.68-1.44) | | |
| Trifluralin | 7814 (53) | | | 56 (54) | | 1.31 (0.86-1.98) | | |
| Phenoxy |  | | |  | |  | | |
| 2, 4-D | 12048 (80) | | | 80 (75) | | 0.88 (0.56-1.39) | | |
| 2, 4, 5-T | 4854 (33) | | | 31 (28) | | 0.88 (0.58-1.36) | | |
| Thiocarbamate | |  |  | |  | |  |  |
| Butylate | 4972 (34) | | | 36 (34) | | 1.04 (0.67-1.62) | | |
| EPTC | 2474 (17) | | | 20 (19) | | 1.48 (0.89-2.46) | | |
| Triazine/Triazinone |  | | |  | |  | | |
| Atrazine | 10977 (75) | | | 83 (78) | | 1.31 (0.79-2.14) | | |
| Cyanazine | 6005 (41) | | | 43 (40) | | 1.28 (0.83-1.96) | | |
| Metribuzin | 6866 (46) | | | 51 (47) | | 1.36 (0.90-2.04) | | |
| Other |  | | |  | |  | | |
| Chlorimuron_Ethyl | 5151 (35) | | | 39 (36) | | 1.16 (0.78-1.72) | | |
| Dicamba | 7332 (50) | | | 48 (45) | | 1.13 (0.72-1.80) | | |
| Glyphosate | 11997 (79) | | | 87 (81) | | 1.13 (0.70-1.83) | | |
| Imazethapyr | 5810 (40) | | | 35 (33) | | 1.02 (0.63-1.65) | | |
| Paraquat | 3419 (23) | | | 28 (26) | | 1.00 (0.63-1.57) | | |
| Petroleum oil/ distillates | 6745 (46) | | | 54 (48) | | 1.31 (0.89-1.93) | | |
| **Fungicides and Fumimgants** |  | | |  | |  | | |
| Benomyl | 1729 (12) | | | 23 (21) | | 1.75 (1.05-2.90) | | |
| Captan | 1472 (10) | | | 13 (12) | | 1.34 (0.75-2.40) | | |
| Chlorothalonil | 1176 (8) | | | 14 (13) | | 1.00 (0.50-1.98) | | |
| Maneb | 1618 (11) | | | 15 (14) | | 1.02 (0.57-1.82) | | |
| Metalaxyl | 3370 (23) | | | 36 (33) | | 1.42 (0.91-2.23) | | |
| Methylbromide | 2342 (15) | | | 23 (21) | | 1.14 (0.67-1.93) | | |

FFS = Fee for Service; DDT-Dichlorodiphenyltrichloroethane; EPTC-S-Ethyl dipropylthiocarbamate.

**^1^**Lifetime history ever used specific pesticides at enrollment (1993-1997) or first follow-up (1999-2003) prior to rheumatoid arthritis (RA) diagnosis.

**^2^**Among participants with ≥36 months complete continuous fee for service (FFS) coverage, including an initial ≥24-month clean period with no claims for RA; cases had ≥2 claims for RA ≥30 days apart, plus ≥ 1 claim from a rheumatologist.

**^3^**Relative Risk estimates calculated using log binomial regression-adjusted for categorical age at enrollment (40-49, 50-59, 60-69, 70+), state (NC-IA), education (≤high school->high school), smoking pack years, and correlated pesticides (R2>0.35): aldicarb (chlorothalonil and benomyl)-chlorothalonil (aldicarb and benomyl)-atrazine (alachlor)-butylate (cyanazine and metribuzin).

Supplementary Table 6. Non-cancer reference dose (RfD) for chronic oral exposure and human health systems affected: specific pesticides associated with RA risk among AHS applicators with ≥24 months continuous Medicare-FFS (1999-2016).

| **Pesticide**  (year introduced) | **Reference dose**^1^: Mg/kg/day  System (certainty) | **Human Systems**^2^ |
| --- | --- | --- |
| Malathion  (1952) | 1 x 10 ^-2^  Nervous (medium) | Endocrine disruptor, neurological, reproductive |
| Phorate  (1959) | 5 x 10 ^-4^  Nervous/hematologic | Neurological |
| Carbaryl  (1957) | 1 x 10 ^-1^  Kidney/Liver (medium) | Endocrine disruptor, reproductive, developmental, neurological |
| Carbofuran  (1965) | 5 x 10 ^-3^  Nervous, reproductive (high) | Endocrine disruptor, reproductive, developmental |
| Alachlor  (1969) | 1 x 10 ^-2^  Hematologic (high) | (possible endocrine disruptor, lung, kidney) |
| Metolachlor  (1976) | 1.5 x 10 ^-1^  Reproductive/other (high) | Endocrine disruptor |
| S-Ethyl dipropylthiocarbamate  (1957) | 2.5 x 10 ^-2^  Cardiomyopathy (medium) | Neurological |
| Metribuzin  (1968) | 2.5 x 10 ^-2^  Hepatic, urinary, other (medium) | Endocrine disruptor, reproductive/developmental |
| Benomyl  (1968) | 5 x 10 ^-2^  Developmental (high) | Endocrine disruptor, reproductive, respiratory |

^1^Based on Integrated Risk Information System (IRIS), United States Environmental Protection Agency (including certainty level; [Integrated Risk Information System | US EPA](https://www.epa.gov/iris), accessed Sept 9, 2024); or other source, i.e., phorate, Human Health Assessment Scoping Document, EPA (1/21/2009).

^2^All are listed as Cramer Class III, indicating high probability of toxicity, table specifically lists human health systems impacted, except skin and eye; malathion, phorate, carbaryl, carbofuran, and S-Ethyl dipropyl thiocarbamate toxicity include acetylcholinesterase inhibition. Pesticide Properties Database, University of Hertfordshire, U.K. (<https://sitem.herts.ac.uk/aeru/ppdb/en/> - accessed Sept 9, 2024).
